# The Conditions Matter: The Toxicity of Titanium Trisulfide Nanoribbons to Bacteria *E. coli* Changes Dramatically Depending on the Chemical Environment and the Storage Time

**DOI:** 10.3390/ijms24098299

**Published:** 2023-05-05

**Authors:** Olga V. Zakharova, Valeria V. Belova, Peter A. Baranchikov, Anna A. Kostyakova, Dmitry S. Muratov, Gregory V. Grigoriev, Svetlana P. Chebotaryova, Denis V. Kuznetsov, Alexander A. Gusev

**Affiliations:** 1 Institute for Environmental Science and Biotechnology, Derzhavin Tambov State University, 392020 Tambov, Russia; olgazakharova1@mail.ru (O.V.Z.); vuz.lera@gmail.com (V.V.B.); petrovi4-98@yandex.ru (P.A.B.); anya.kostyakova2014@yandex.ru (A.A.K.); bboykick@outlook.com (G.V.G.); sweta-chebotarjova@yandex.ru (S.P.C.); 2 Department of Functional Nanosystems and High-Temperature Materials, National University of Science and Technology «MISIS», 119991 Moscow, Russia; muratov@misis.ru (D.S.M.); dk@misis.ru (D.V.K.); 3 Engineering Center, Plekhanov Russian University of Economics, 117997 Moscow, Russia; 4 Scientific School “Chemistry and Technology of Polymer Materials”, Plekhanov Russian University of Economics, Stremyanny Lane 36, 117997 Moscow, Russia

**Keywords:** TiS_3_ nanoribbons, TiO_2_ nanoparticles, H_2_S, non-linear toxicity, antibacterial activity, bioluminescent strain of *E. coli*, dispersion medium, storage time

## Abstract

In this work, we present an analysis of the antibacterial activity of TiS_3_ nanostructures in water and 0.9% NaCl solution suspensions. TiS_3_ nanoribbons 1–10 µm long, 100–300 nm wide, and less than 100 nm thick were produced by the direct reaction of pure titanium powder with elemental sulphur in a quartz tube sealed under vacuum. For the toxicity test of a bioluminescent strain of *E. coli* we used concentrations from 1 to 0.0001 g L^−1^ and also studied fresh suspensions and suspensions left for 24 h. The strongest toxic effect was observed in freshly prepared water solutions where the luminescence of bacteria decreased by more than 75%. When saline solution was substituted for water or when the solutions were stored for 24 h it resulted in a considerable decrease in the TiS_3_ antibacterial effect. The toxicity of TiS_3_ in water exceeded the toxicity of the reference TiO_2_ nanoparticles, though when saline solution was used instead of water the opposite results were observed. In addition, we did not find a relationship between the antibacterial activity of water suspensions of nanoribbons and the stability of their colloidal systems, which indicates an insignificant contribution to the toxicity of aggregation processes. In 0.9% NaCl solution suspensions, toxicity increased in proportion to the increase in the zeta potential. We suppose that the noted specificity of toxicity is associated with the emission of hydrogen sulphide molecules from the surface of nanoribbons, which, depending on the concentration, can either decrease or increase oxidative stress, which is considered the key mechanism of nanomaterial cytotoxicity. However, the exact underlying mechanisms need further investigation. Thus, we have shown an important role of the dispersion medium and the period of storage in the antibacterial activity of TiS_3_ nanoribbons. Our results could be used in nanotoxicological studies of other two-dimensional nanomaterials, and for the development of novel antibacterial substances and other biomedical applications of this two-dimensional material.

## 1. Introduction

In recent years, promising anisotropic electrical and optical properties of layered materials have been revealed [1]. Harnessing the anisotropic properties of these materials has resulted in various applications, such as an integrated digital inverters [2] and linear-dichroic photodetectors [3]. It was also shown that transition metal dichalcogenides make good bio-visualization [4], drug delivery [5,6], and phototherapy agents [7] as well as biosensors [8].

Transition metal trichalcogenides are a prospective family of materials that combine the advantages of 2D materials (flexibility, transparency, and others) with fully quasi-one-dimensional (1D) properties [9,10,11,12]. In particular, titanium trisulfide (TiS_3_) is a 2D semiconductor belonging to the MX3 family that possesses quasi-1D properties and has potential for various applications in the future, including biomedical ones [13,14].

The growing use of two-dimensional transition metal chalcogenides contributes to an increase in their accumulation in the environment and the possibility of affecting living objects, including humans. In addition, the use of two-dimensional transition metal chalcogenides in biomedicine involves their direct injection into the body, which will lead to accumulation in human organs and tissues [15]. Thus, the assessment of the biosafety of such materials is an important step in the development of materials and devices based on them.

For example, for transition metal dichalcogenides (MoS_2_, WS_2_, and WSe_2_), MoS_2_ and WS_2_ nanosheets have been shown to cause low toxicity to A549 cells (human lung carcinoma epithelial cells), even at high concentrations. At the same time, WSe_2_ had a dose-dependent cytotoxic effect, reducing cell viability by up to 31.8% at a maximum concentration of 400 µg mL^−1^ [16]. In [17], using combined experimental and theoretical approaches, the authors compared the cytotoxic effects of MoS_2_ nanosheets with a 5-layer and a 40-layer structure. It has been shown that at a concentration determined by the nanosheet surface area (10 cm^2^ mL^−1^), 40-layer nanosheets are internalized by cells, while 5-layer nanosheets mainly bind to the cell surface without internalization. Although, depending on the structure, different nanomaterials alter different genes associated with autophagy, the common mechanism is that they disrupt amyloid precursor proteins of cell surface proteins and activate the mTOR signalling pathway. Similar results were obtained for WS_2_ nanoplates [18]. Other authors have also shown the influence of the thickness of MoS_2_ plates on their cytotoxicity [19]. An increase in the toxicity of MoS_2_ towards human lung cells with a decrease in the thickness of the nanoplates was revealed. The authors suggest that this may be due to an increase in surface area and active edge sites. MoS_2_ accelerated proliferation, the initiation of myogenic differentiation, and epithelial–mesenchymal transition in human embryonic lung fibroblasts. Abnormal proliferation and differentiation contributed to idiopathic pulmonary fibrosis. MoS_2_ also significantly stimulated the expression of genes and proteins associated with myofibroblasts and mesenchymal cells [20]. MoS_2_ nanoplates reduced the viability of human hepatoma cells at 30 µg mL^−1^, caused ROS generation (≥2 µg mL^−1^) and mitochondrial depolarization (≥4 µg mL^−1^), and disrupted membrane integrity at concentrations ≥8 µg mL^−1^ [21].

At the same time, an evaluation of the effect of 2D materials on human mesenchymal stem cells derived from adipose tissue did not reveal an acute toxicity of molybdenum sulphide (MoS_2_) or tungsten sulphide (WS_2_) in the low concentration range (<5 µg mL^−1^). Interestingly, the substrates modified with 2D materials showed higher cell adhesion, expansion, and proliferation compared to the untreated substrate. In the case of differentiation, substrates modified with MoS_2_ or WS_2_ showed better efficiency in terms of controlling cell adipogenesis compared to an untreated substrate [20].

Despite a significant number of studies on the toxicity of two-dimensional transition metal dichalcogenides, there are very few studies on the biological effects of transition metal trichalcogenides. Our previous studies show the effectiveness of 2D ZrS_3_ and TiS_3_ nanoribbons as sterilizing agents, growth stimulators, and activators of rhizogenesis of micro-sprouts of tree crops during clonal micropropagation [22]. TiS_3_ nanoribbons of thickness less than 100 nm, length 1–10 µm, and width 0.4–1 µm showed promise for use as a sterilizing and stimulating agent in the initial growth stage and as a rhizogenesis-activating agent in the rooting stage of poplar × aspen hybrid and downy birch microclones [23].

In addition, we demonstrated that freshly prepared suspensions of ZrS_3_ nanoplates in saline at concentrations up to 1 g L^−1^ had no toxic effect on E. coli bacteria; however, ZrS_3_ suspensions that were stored for 24 h were very toxic to bacteria even at concentrations up to 0.001 g L^−1^ [24].

Since there is still no clear understanding of the specifics and mechanisms of the influence of the chemical environment on the toxicity of 2D transition metal trichalcogenides, the aim of the present study is to carry out an evaluation of the toxicity of TiS_3_ nanoribbons on *E. coli* bacteria depending on the composition of the media and the storage time of the suspension.

## 2. Results

### 2.1. TiS_3_ Nanoribbons Characterization

**Figure 1:**
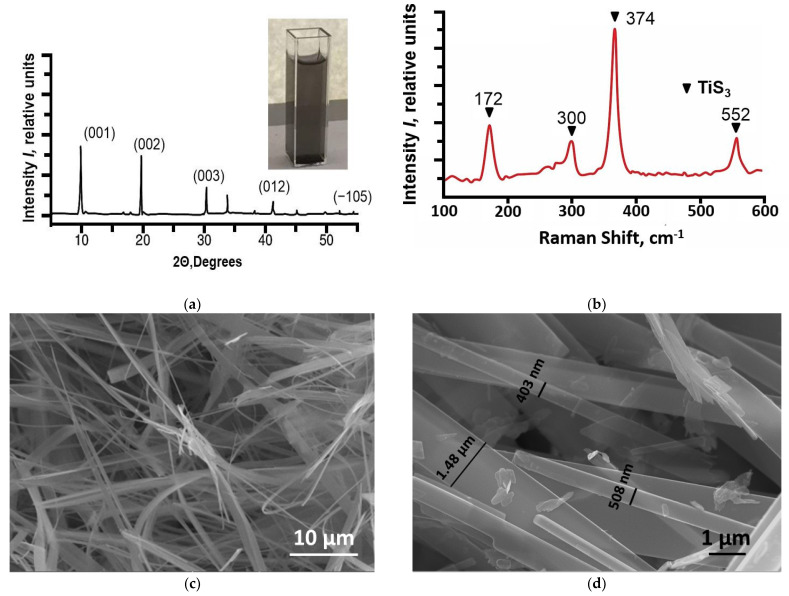
TiS_3_ characterization: (**a**) XRD spectra and appearance of a TiS_3_ dispersion; (**b**) Raman spectra; (**c**,**d**) SEM images.

Figure 1 presents the TiS_3_ nanoribbons characterization.

The results of the X-ray diffraction analysis of a TiS_3_ sample are presented in Figure 1a, one can see that all the main lines correspond to the TiS_3_ phase [25,26]. The radiographs were recorded in the range 0–60° 2θ and assigned from the PDF-2 database file compiled by the International Diffraction Center. The phase content was determined by the Rietveld method in the TOPAS software. Raman spectroscopy (Figure 1b) showed prominent TiS_3_ bands [27,28], thus proving that this phase was present in the sample. 

Scanning electron microscopy (Figure 1c,d) showed that the average length was more than 10 µm and width was from 0.3 to 2 µm. The thickness of the obtained TiS_3_ nanoribbons ranged under 100 nm [23].

### 2.2. Antibacterial Properties

**Figure 2:**
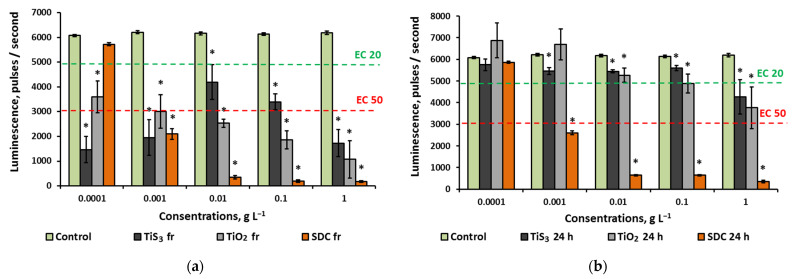
Antibacterial properties of TiS_3_ nanoribbons aqueous dispersions: (**a**) fresh; (**b**) 24 h. Abbreviations: SDC—sodium dichloroisocyanurate; EC20—the effective concentration of the sample causing a 20% quenching of the biosensor glow compared to the control; EC50—the effective concentration of the sample causing a 50% quenching of the biosensor glow compared to the control; values significantly differing from the control are marked with an asterisk *.

Figure 2 presents the results of an antibacterial properties assessment of aqueous suspensions of titanium-containing substances.

**Figure 3:**
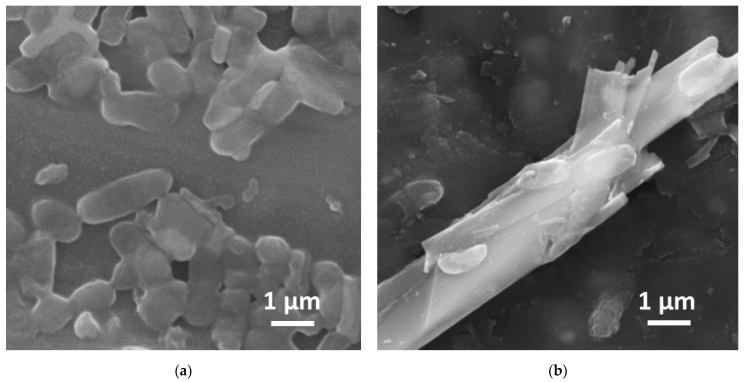
SEM images of *E. coli*: (**a**) before and (**b**) after exposure with TiS_3_ freshly prepared water solution (1 g L^−1^).

The TiS_3_ sample in freshly prepared suspensions showed a strong toxic effect in every studied concentration though the dose/toxicity correlation was nonlinear (Figure 2a). The highest inhibiting effect was recorded at 0.0001 and 1 g L^−1^ where the decrease in luminescence was more than 75%, while the concentration of 0.01 g L^−1^ had the weakest effect with luminescence suppressed by 30%. Nonlinear dose–effect relationships have been revealed in several previous studies of various nanoparticles; this phenomenon can be associated with aggregation processes in colloidal solutions [29], bio-objects’ adaptation [30], or signalling effects produced by nano-objects. The effect of 24 h TiS_3_ suspensions against *E. coli* was much weaker (Figure 2b), and the non-linear nature of toxicity was levelled. The strongest antibacterial activity was registered at 1 g L^−1^ with luminescence inhibited by 30%. SEM images of *E. coli* before and after exposure with TiS_3_ at maximum concentration are shown in Figure 3. 

As can be seen from the SEM image (Figure 3b), bacteria are partially degraded in the presence of TiS_3_ nanoribbons.

Such a decrease in the antibacterial properties of the aged nanoparticles suspensions can be explained by their aggregation [31]. Moreover, free sulphur can be released from the nanoribbons’ surface, resulting in the formation of hydrogen sulphide. In freshly prepared suspensions, its concentration may exceed safe values and have a toxic effect, while over time, the concentration of H_2_S decreases [23]. It is known that H_2_S in physiological concentrations can protect cells against oxidative stress [32], which is the main toxicity mechanism of nanoparticles [33,34,35].

Analysis of TiO_2_ antibacterial properties revealed that, unlike TiS_3_, TiO_2_ dispersions showed a linear dose-dependent effect. The minimum luminescence of bacteria was observed at the highest concentration and amounted to 17% of the control value for fresh solutions and 62% for daily solutions. 

The control sample of SDC dose-dependently inhibited bacterial growth at a concentration as low as 0.001 g L^−1^, while at 0.01–1 g L^−1^ the bacterial survival rate was close to zero. Storage time had almost no influence on the efficacy.

**Figure 4:**
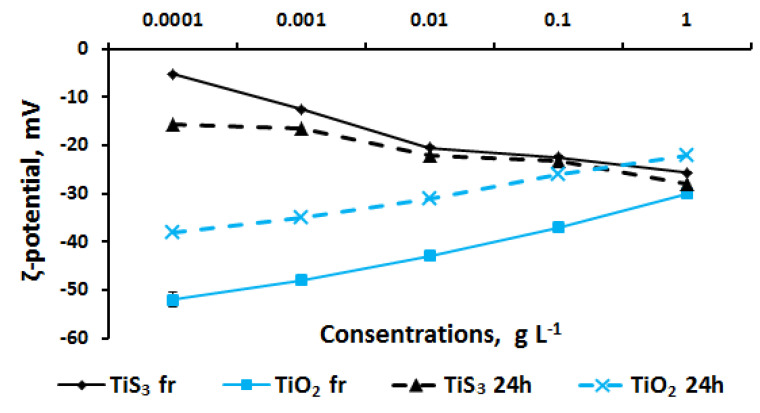
Stability of TiS_3_ and TiO_2_ aqueous dispersions.

The study of the stability of TiS_3_ aqueous dispersions revealed a correlation between zeta potential value and concentration (Figure 4). TiS_3_ colloids with the highest concentration were more stable than the low-concentration dispersions and the storage time had an insignificant effect on this characteristic. For aqueous TiO_2_ colloids, an inverse relationship was noted: with increasing concentration, the stability of dispersions decreased. Daily storage partially reduced the zeta potential.

Upon comparison, the results of toxicological studies and the stability of the TiS_3_ dispersion system revealed no evident correlations. At the same time, TiO_2_ suspensions increased toxicity with a decrease in the stability index. 

**Figure 5:**
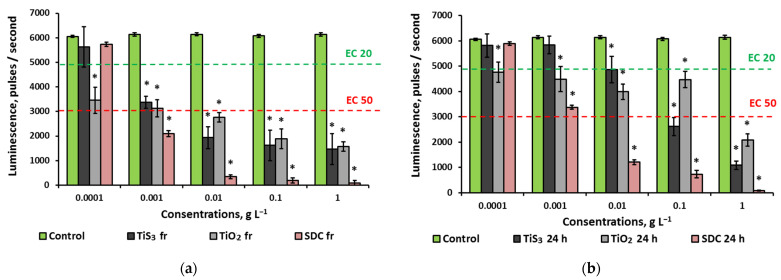
Antibacterial properties of TiS_3_ in saline solution: (**a**) fresh; (**b**) 24-h, values significantly differing from the control are marked with an asterisk *.

When saline solution was used as a dispersion medium, a significant change in the antibacterial properties of the studied substances was observed (Figure 5a,b). The non-linear toxic effects, observed in aqueous media, were replaced by linear ones. The maximum inhibition of bacterial luminescence was observed at 1 g L^−1^—the decrease in the indicator was 76%. When the concentration decreased to 0.001 g L^−1^, the toxicity also decreased (luminescence intensity 60% relative to control), while at 0.0001 a zero antibacterial activity was registered. The 24 h TiS_3_ dispersions had a reduced effect: at 0.001 g L^−1^ no growth suppression was observed and at 0.01 and 0.1 g L^−1^ the efficacy fell nearly by half.

Fresh TiO_2_ suspensions in saline solution showed a linear toxicity/concentration relationship (Figure 5a). The highest antibacterial effect was registered at 1 g L^−1^ (luminescence reduction by 75%) when the solution was diluted to 0.0001 g L^−1^, luminescence decreased by 40% relative to the control. After 24 h storage, the 0.0001–0.1 g L^−1^ dispersions lost most of their toxicity while the antibacterial effect of the 1 g L^−1^ dispersion changed very little. 

Some alteration in the antibacterial properties of the control samples in saline solution was also observed, when the toxic activity was registered in solutions with concentrations from 0.01 g L^−1^ and upwards, while aqueous dispersions started displaying antibacterial activity at 0.001 g L^−1^. Storage time had a negligible effect on the toxicity of the control samples. 

It is possible that the toxicity mechanism of TiS_3_ can be attributed to oxidative stress, similar to that produced by TiO_2_ nanoparticles [36,37] or MoS_2_ nanosheets [38]. This effect can be increased by hydrogen sulphide formed when sulphur is released into the solution from the nanoribbons’ surface, as in high concentrations hydrogen sulphide ceases to protect cells and starts inhibiting antioxidant enzymes in the process of induced oxidative stress [39]. Besides that, the antibacterial effect can be attributed to direct mechanical damage caused by TiS_3_ nanoribbons to bacterial cells, similar to that shown by graphene-like materials [40].

**Figure 6:**
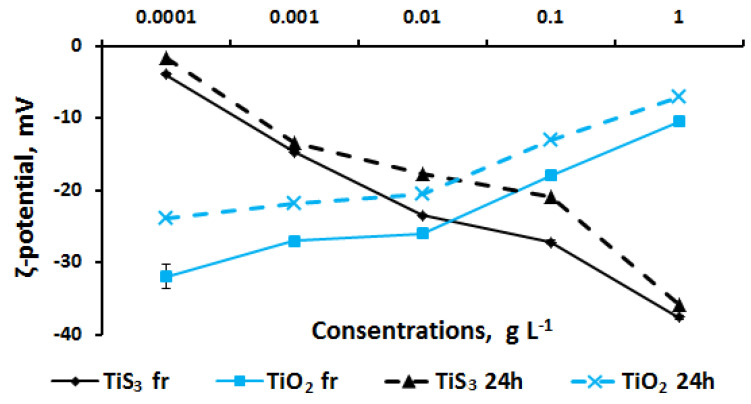
Stability of TiS_3_ and TiO_2_ in saline solution.

Stability analysis of TiS_3_ dispersions in saline solution revealed the dependency between the zeta potential and substance concentration (Figure 6). In general, in saline solutions, a considerable increase in the stability of high-concentration (1 g L^−1^) solutions can be noted. Suspensions of TiO_2_, as in an aqueous medium, showed a decrease in the zeta potential with an increase in the concentration of nanoparticles. At the same time, the replacement of the aqueous dispersion medium with a saline solution resulted in a general decrease in the stability of colloidal systems. For example, in water at 0.0001 g L^−1^, the ζ-potential value was −52 mV for a fresh suspension and −38 mV for a daily suspension; in saline, this indicator was −32 mV and −24 mV, respectively. 

Upon comparison of the results of toxicological studies and the stability of dispersed systems in a saline solution, it can be seen that the toxicity increased linearly to the values of the zeta potential for TiS_3_ solutions and inversely proportionally for TiO_2_ dispersions.

**Figure 7:**
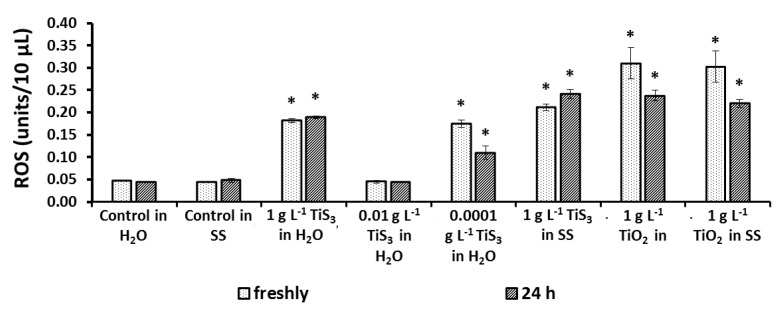
Intracellular ROS production (SS—saline solution), values significantly differing from the control are marked with an asterisk *.

An oxidative stress test was carried out to determine the mechanisms by which nanomaterials affect bacteria at the most toxic concentrations (Figure 7).

In all cases where the luminescence of bacteria was significantly reduced, the increase in ROS was noted (Figure 2 and Figure 5). At the same time, in the case of an average concentration of TiS_3_ in the aquatic environment, where a low degree of toxicity was observed, the level of oxidative stress was at the level of control indicators, which, as we discuss below, may be associated with the protective effect of hydrogen sulphide at certain concentrations.

Additionally, we analysed the content of hydrogen sulphide in the studied solutions. We have found that the content of H_2_S increases with the concentration of TiS_3_ in both fresh and 24 h aqueous suspensions. At the same time, the appearance of sodium chloride in the medium affected the content of hydrogen sulphide in different directions. In all cases, the content of H_2_S in the medium decreased significantly over time. The results of this experiment are presented in Table 1.

Thus, we were the first team to study the antibacterial activity of TiS_3_ nanoribbons on *E. coli*. This research has revealed a significant association between the storage time and dispersion medium of the suspensions and their antibacterial properties and stability. The strongest toxic effect was shown by freshly prepared aqueous TiS_3_ colloid solutions with a decrease in luminescence by 75%. The antibacterial efficacy of TiS_3_ decreases dramatically when saline solution is used instead of distilled water and when the suspensions are stored for 24 h. In water, the TiS_3_ toxicity exceeds that of TiO_2_, though replacing water with saline solution as the dispersion medium leads to opposite results. 

The zeta potential measurement revealed no evident correlation between the water colloids’ stability and toxicity rate. The toxicity of TiS_3_ nanoribbons can be attributed to oxidative stress, direct mechanical damage to bacterial cells, or exposure to hydrogen sulphide, though this topic requires further research. 

Therefore, we showed an important role of the disperse medium and storage time in the antibacterial activity of TiS_3_ nanoribbons. 

## 3. Discussion

It turned out that aqueous suspensions of TiS_3_ nanoribbons exhibit non-linear toxicity, maximally suppressing bacterial luminescence at the lowest (0.0001 g L^−1^) and highest (1 g L^−1^) nanomaterial concentrations in a freshly prepared solution. At the same time, suspensions of TiO_2_ nanoparticles, taken for comparison, had a dose-dependent antibacterial effect, but this was weaker than that of TiS_3_. An analysis of the toxic effect and stability of TiS_3_ dispersions did not reveal any relationship between these parameters. After daily storage, the bactericidal effect of TiS_3_ and TiO_2_ solutions decreased, and the non-linear toxicity practically disappeared. 

The high toxicity of freshly prepared solutions of TiS_3_, as mentioned earlier, may be associated with the release of H_2_S [23], which can inhibit the work of antioxidant enzymes of the cell under conditions of oxidative stress caused by nanoparticles [39]. However, it was also observed that hydrogen sulphide evaporated from the solution over time [23]. The decrease in the content of hydrogen sulphide in titanium trisulfide colloids within 24 h was confirmed by us by direct measurements. This explains the loss of the antibacterial effect of the titanium trisulfide 24 h solutions.

Evidence of the bactericidal action of hydrogen sulphide is presented in the work in [41]. The authors found that the antibacterial property of hydrogen sulphide is based on oxidative stress. H_2_S released by sodium hydrosulphide (NaHS) significantly inhibits the growth of *E. coli* in a dose-dependent manner. Further studies have shown that hydrogen sulphide treatment stimulates the production of reactive oxygen species (ROS) and reduces glutathione levels in *E. coli* cells, leading to lipid peroxidation and DNA damage. H_2_S also inhibits the activity of antioxidant enzymes (superoxide dismutase, catalase, and glutathione reductase). 

However, there is evidence of a positive effect of hydrogen sulphide on bacteria. So, in the study in [32], it is stated that hydrogen sulphide in physiological concentrations protects cells from oxidative stress and antibiotics using two mechanisms: (1) elimination of DNA breaks and (2) increased activity of catalase and superoxide dismutase. It was shown in [42] that the biological effects of H_2_S are two-phase, passing from cytoprotection (at low, nanomolar concentrations) to cytotoxicity due to an increase in the concentration of the compound. Endogenously generated H_2_S increases the resistance of bacteria to oxidative stress caused by antibiotics. Reactive oxygen species induced by antibiotics lead to DNA damage via the Fenton reaction. It is assumed that H_2_S prevents oxidative damage to bacterial DNA through the following cytoprotective mechanisms: (1) direct conversion of H_2_O_2_ to H_2_O; (2) decrease in the concentration of Fe^2+^, the catalyst for the Fenton reaction; (3) a decrease in the content of free cysteine, which is a reducing agent in the Fenton reaction; and (4) stimulation of the activity of superoxide dismutase and catalase.

Thus, hydrogen sulphide in physiological concentrations protects cells from oxidative stress. With an increase in the content of hydrogen sulphide, the antibacterial effect associated with oxidative stress increases. Therefore, the nonlinear toxicity of suspensions of TiS_3_ nanoribbons can be explained as follows: At a concentration of 0.0001 g L^−1^, the content of hydrogen sulphide is below physiological values, which leads to oxidative stress caused by TiS_3_ nanoribbons. This is confirmed in the work in [43]: the low concentration of H_2_S (~10 mM) precludes its involvement in counteracting oxidative stress. Further, in our experiment, the second increase in TiS_3_ toxicity was observed at a concentration of 1 g L^−1^. This rise is due to the fact that the content of hydrogen sulphide probably exceeded physiological values; therefore, this contributed to the development of oxidative stress.

When replacing water with saline, completely different effects were noted. First of all, in the saline medium, the antibacterial effect of TiS_3_ increased in proportion to the growth of the nanomaterial concentration, and, in contrast to the aqueous medium, no significant inhibition of luminescence was observed at the minimum concentration (0.0001 g L^−1^). In addition, the toxicity of TiO_2_ suspensions in saline was higher than that of TiS_3_ dispersions. In our experiment, the toxicity of TiS_3_ was lower in saline (0.9% NaCl solution) compared to the aqueous medium. This can be explained by the fact that the composition of the saline solution includes mineral elements necessary for the life of *E. coli*. We did not obtain confirmation of a decrease in the emission of hydrogen sulphide from TiS_3_ nanoribbons in a saline medium compared to an aqueous one (Table 1); therefore, it remains to assume a stress-protective role of sodium chloride. According to other authors, NaCl ions can indeed increase the oxidative resistance of bacterial cells via chloride and sodium ion channels rather than the degradation of hydrogen peroxide [44]. At the same time, although the optimal content of sodium chloride in water for *E. coli* is 0.5%, these bacteria can successfully adapt to an 11% solution [42], so the 0.9% solution used can be considered physiological for bacteria. However, the exact molecular mechanisms that affect the toxicity of TiS_3_ nanoribbons in various media require additional research.

At the same time, the antibacterial efficacy of TiO_2_ nanoparticles, which we used as a reference nanotoxicant, was dose-dependent in all cases, in contrast to TiS_3_. A factor that reduces toxicity was the daily storage of colloids. It is known that TiO_2_ nanoparticles have antibacterial activity associated with UV photooxidation, although they retain antibacterial properties without UV radiation [45]. As specific molecular and cellular mechanisms of toxicity, the following are considered: (a) ROS production and the formation of electron–hole pairs in the presence of light; (b) the binding of TiO_2_ nanoparticles to the cell membrane through electrostatic interactions, which leads to cell wall damage and lipid peroxidation in the cell membrane; and (c) cytoplasmic leakage and attachment of TiO_2_ nanoparticles to intracellular organelles and biological macromolecules after cell membrane damage [46]. An important factor affecting the toxicity of titanium dioxide nanoparticles is their size [47]. Storage of TiO_2_ suspensions promotes the aggregation of nanoparticles, while fresh suspensions contain smaller nanoparticles [48]. Some authors note a significant decrease in the negative ζ potential of TiO_2_ nanoparticles in seawater suspensions compared to ultrapure water, probably due to the high concentration of ions, which suppresses the electrostatic repulsion of negative charges, which can accelerate homoaggregation [49]. Similar results were obtained in our experiment. 

## 4. Materials and Methods

The TiS_3_ sample studied in this work was obtained by gas-phase synthesis in vacuum [9]. At the first stage of TiS_3_ synthesis, precisely weighed quantities of pure (99.99%) titanium and analytical-reagent-grade elemental sulphur powders were prepared in stoichiometric proportion according to reaction (1).

Ti + 3S = TiS_3_ (1)

Thus, the obtained sample was analysed using a Thermo Scientific DXR Raman microscope (Waltham, MA, USA) with a 532 nm laser at 1 mW power through a 100× objective, X-ray diffraction analysis (D2 Phaser, Bruker AXS, Karlsruhe, Germany) and scanning electron microscope JCM-7000 NeoScope (JEOL Ltd., Akishima-shi, Japan).

For measuring antibacterial properties of TiS_3_ we used a lyophilized culture of luminescent *Escherichia coli* (Immunotech, Moscow, Russia) [24,50]. The criterion of toxicity is the change in the intensity of the bioluminescence of the test object in the test sample compared to the control, which does not contain toxic substances. The decrease in the intensity of bioluminescence is proportional to the toxic effect [51]. The toxic effect of the studied nanomaterial sample on bacteria is determined by the inhibition of bioluminescence over a 30 min exposure period.

The measurements were carried out on a portable Biotox-10 device (Nera S, Moscow, Russia). 

For the present study, dispersions of TiS_3_ nanoribbons were prepared in distilled water (pH 7.1 ± 0.2) and in saline solution (SS)—0.9% NaCl (pH 7.1 ± 0.2). Quantities of nanoribbons (100 mg) were poured into the prepared dispersion medium (100 mL) and stirred with a glass rod for 2–3 min. After stirring, the suspensions were processed in Ultrasonic Cleaner CD-4800 (Codyson, Shenzhen, China), 3 min treatment cycle was repeated four times, the suspension was stirred with the glass rod after each cycle. The initial suspension had particle concentration of 1 g L^−1^, it was diluted with distilled water or saline solution in order to prepare suspensions with particle concentrations of 0.1–0.0001 g L^−1^. 

For comparison, dispersions of titanium dioxide (TiO_2_) nanoparticles (Sigma-Aldrich, St. Louis, MO, USA) (average size 30–40 nm) were analysed because many 2D materials, including TiS_3_, suffer from oxidation and degradation effects under ambient conditions [52,53,54]. TiO_2_ suspensions were prepared similarly to TiS_3_ suspensions.

Similar concentrations of sodium dichloroisocyanurate solutions (SDC) (NPF Praktika LLC, Yoshkar Ola, Russia) were employed for positive control. This product is used as a disinfectant active against Gram-positive and -negative bacteria, viruses, Candida fungi, and dermatophytes.

Influence of storage time on antibacterial properties of nanoparticle colloid solutions was analysed using fresh (0.5 h or less) and 24 h colloids.

The study was carried out in 5 biological and 3 analytical replications.

Reactive oxygen species (ROS) generated intracellularly as a result of exposure to nanomaterials on *E. coli* were measured using the nitro blue tetrazolium reduction assay (NBT) [55]. An amount of 100 µL of bacterial suspension (OD600 = 1.5) was incubated with suspensions of nanoparticles that had the greatest toxic effect in the bioluminescent test and 500 µL of 1 mg mL^−1^ NBT for 1 h at 37 °C. Then, 100 µL of 0.1 M HCl was added and the tubes were centrifuged at 1500× *g* for 10 min. The pellet was treated with 600 µL of dimethyl sulfoxide (DMSO) to lyse the cells and recover the reduced NBT. The measurements were carried out on a Multiskan Sky spectrophotometer (Thermo Scientific, Waltham, MA, USA) at a wavelength of 575 nm.

H_2_S concentrations in water and saline solutions were determined by iodometric method [56,57]. The method is based on the oxidation of hydrogen sulphide and its associates in an acidic environment with iodine taken in excess. The amount of iodine consumed for oxidation is determined by the difference between the added amount of iodine and its excess, which is titrated with thiosulfate. In this study, we analysed fresh and 24 h solutions containing 0.0001, 0.01, and 1 g L^−1^ TiS_3_.

Zeta potential (ζ-potential) was measured using Zetasizer NanoZS (Malvern, UK) in order to evaluate effect of suspensions’ stability on their toxicological characteristics. Zeta potential is the electric potential created between the charged groups associated with the surface of a particle and the suspension medium and can be used to derive information concerning the particle surface charge [58]. As cell membranes are negatively charged, the degree of interaction between a particle and a cell surface or the membrane of an organelle may be influenced by the ζ-potential of a nanoparticles in contact with it [59].

The employed descriptive statistics methods included evaluation of the arithmetic mean (M) and mean-square deviation (S) calculated in Excel 2007 (MS Office 2007, Redmond, WA, USA). Fisher criterion was employed to determine statistical significance of the variations between the qualitative variables of the studied groups [60].

The research was carried out using the equipment of the Center for Collective Use of Scientific Equipment of TSU named after G.R. Derzhavin.

## 5. Conclusions

We found that the chemical composition of the medium and the storage time of the suspension are important factors that can affect experiments evaluating the toxicity of titanium trisulfide nanoribbons on *E. coli* bacteria. The highest toxicity in our experiment was exhibited by a freshly prepared colloidal solution of TiS_3_ nanoribbons in an aqueous medium. The antibacterial effect of TiS_3_ nanoparticles decreased in physiological saline, as well as when keeping the suspension for 24 h. A colloidal solution of TiS_3_ nanoribbons in an aqueous medium was characterized by a non-linear dose–effect dependence; however, in a saline solution, this dependence changed to a linear one. It could be assumed that the antibacterial effects are associated with the formation of a titanium dioxide film on the surface of TiS_3_ nanoribbons as a result of oxidation, but our control experiment revealed a completely different characteristic of the antibacterial effect of TiO_2_ nanoparticles. In addition, we did not find a relationship between the antibacterial activity of nanoribbons and the stability of their colloidal systems, which indicates an insignificant contribution to the toxicity of aggregation processes. Therefore, we associate the noted antibacterial effects of TiS_3_ in distilled water with the emission of hydrogen sulphide from the surface of nanoribbons, which at physiological concentrations is an inhibitor of oxidative stress, the main damaging mechanism of nanotoxicity, and at high concentrations is itself cytotoxic. The emission of hydrogen sulphide decreases over time, and it leaks into the atmosphere, which may cause a decrease in the toxicity of 24 h solutions. In an aqueous medium containing sodium and chloride ions, the formation of hydrogen sulphide was also observed, but, probably, the content of inorganic sodium and chloride ions in the medium in physiological concentrations increases the stress resistance of bacterial cells. Thus, we have shown that the toxicity of colloidal solutions of TiS_3_ nanoribbons significantly depends on the chemical composition of the dispersion medium and their storage time, which is consistent with the data we obtained for other two-dimensional nanomaterials [61,62]. However, the exact underlying mechanisms need further investigation. Our results can be used in nanotoxicological studies of other two-dimensional materials, as well as in the development of new antibacterial materials and preparations.

## Figures and Tables

**Table 1:** H_2_S content in different sample types.

Sample	H_2_S, µg L^−1^	
	Fresh	24-h
0.0001 µg L^−1^ TiS_3_ in water	51.3 ± 0.3	34.36 ± 0.32
0.01 µg L^−1^ TiS_3_ in water	85.36 ± 0.35	47.56 ± 0.25
1 µg L^−1^ TiS_3_ in water	98.46 ± 0.11	91.63 ± 0.15
0.0001 µg L^−1^ TiS_3_ in saline	71.6 ± 0.2	54.63 ± 0.25
0.01 µg L^−1^ TiS_3_ in saline	85.3 ± 0.3	61.43 ± 0.2
1 µg L^−1^ TiS_3_ in saline	102.43 ± 0.4	85.3 ± 0.35

## Data Availability

The data that support the findings of this study are available from the authors.
